# Exploration on neurobiological mechanisms of the central–peripheral–central closed-loop rehabilitation

**DOI:** 10.3389/fncel.2022.982881

**Published:** 2022-09-02

**Authors:** Jie Jia

**Affiliations:** ^1^Department of Rehabilitation Medicine, Huashan Hospital, Fudan University, Shanghai, China; ^2^National Center for Neurological Disorders, Shanghai, China; ^3^National Clinical Research Center for Aging and Medicine, Huashan Hospital, Fudan University, Shanghai, China; ^4^National Regional Medical Center, Fujian, China; ^5^The First Affiliated Hospital of Fujian Medical University, Fujian, China

**Keywords:** central–peripheral–central, closed-loop rehabilitation, stroke, neuro-immune communication, rehabilitation intervention

## Abstract

Central and peripheral interventions for brain injury rehabilitation have been widely employed. However, as patients’ requirements and expectations for stroke rehabilitation have gradually increased, the limitations of simple central intervention or peripheral intervention in the rehabilitation application of stroke patients’ function have gradually emerged. Studies have suggested that central intervention promotes the activation of functional brain regions and improves neural plasticity, whereas peripheral intervention enhances the positive feedback and input of sensory and motor control modes to the central nervous system, thereby promoting the remodeling of brain function. Based on the model of a central–peripheral–central (CPC) closed loop, the integration of center and peripheral interventions was effectively completed to form “closed-loop” information feedback, which could be applied to specific brain areas or function-related brain regions of patients. Notably, the closed loop can also be extended to central and peripheral immune systems as well as central and peripheral organs such as the brain–gut axis and lung–brain axis. In this review article, the model of CPC closed-loop rehabilitation and the potential neuroimmunological mechanisms of a closed-loop approach will be discussed. Further, we highlight critical questions about the neuroimmunological aspects of the closed-loop technique that merit future research attention.

## Introduction

### Concept and development of the theory of central–peripheral–central closed-loop rehabilitation

Proposed in 2016 (Jia, 2016), the CPC closed-loop rehabilitation theory refers to the assessment and therapy consisting of central rehabilitation methods and peripheral procedures. In this novel rehabilitation model, brain plasticity and rehabilitation efficacy following brain injury can be bidirectionally boosted with positive feedback. Related devices can combine input and output capabilities; for example, in the context of a brain–computer interface (BCI), a “closed loop” often refers to the provision of different kinds of feedback, such as proprioceptive feedback and tactile feedback, to the user through vision or other sensory modalities, but can more generally include feedback through any of the artificial input channels.

Long-term rehabilitation is essential for patients with motor dysfunction following a stroke to enable re-learning of motor function and conversion of motor capacity to daily living (Bernhardt et al., 2020). Motor rehabilitation tools for stroke patients mainly focus on peripheral intervention in early years, which include the traditional four major techniques based on the theory of cortical plasticity, which are the Bobath, Brunnstrom, proprioceptive neuromuscular facilitation, and Rood techniques (Huseyinsinoglu et al., 2012; Chen and Shaw, 2014), and new techniques derived from them, such as occupational therapy, compulsory movement therapy, bilateral interventions, anti-spasticity therapy, biofeedback techniques, and electrical stimulation techniques. However, a Cochrane systematic review (French et al., 2016; Legg et al., 2017) reported that the effectiveness of conventional rehabilitation treatment for motor dysfunction in stroke patients is poor, the quality of clinical studies is low, and the rehabilitation effect in many cases is still not evident after the above treatments. With the progression of medical–industrial integration, attempts were made to rehabilitate patients for whom peripheral interventions were ineffective by directly stimulating neural activity in the brain through a top–down approach. Instead of generating feedback through training of the affected limb, this approach employs various evoked modalities to generate central stimulation of the brain injury area to activate neural activity in the relevant brain regions and promote recovery of the patient’s motor function. The central stimulation modalities currently used for stroke motor dysfunction rehabilitation are mainly non-invasive stimulation, including transcranial direct current stimulation (tDCS), transcranial magnetic stimulation (TMS), mirror therapy (MT), mental imagery (MI), BCI, and transcranial ultrasound stimulation (TUS). Systematic reviews have reported that non-invasive stimulation is effective in improving motor function and daily activities in stroke patients, but its mechanism of action remains controversial (Kang et al., 2016).

**FIGURE 1:**
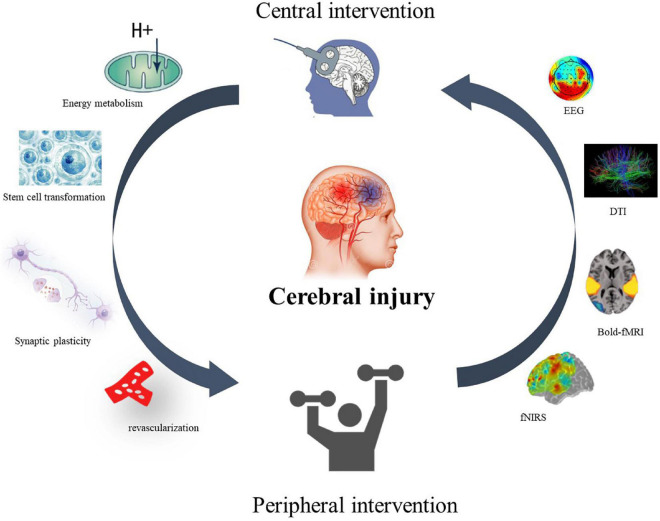
Closed-loop rehabilitation theory. A central–peripheral closed-loop intervention model for motor dysfunction after stroke. The therapeutic effect and mechanism can be reflected by EEG, neuroimaging, cerebral blood oxygen content, synaptic remodeling, energy metabolism, and stem cell transformation.

Neither top–down nor bottom–up interventions can create a closed-loop effect of stimulation for patients’ rehabilitation. However, the CPC treatment model proposed by the team in 2016 theoretically suggests a closed-loop rehabilitation of CPC for motor dysfunction in stroke (Jia, 2016). The closed-loop rehabilitation theory (Figure 1) refers to the combination of the aforementioned central interventions with peripheral interventions to form a positive feedback loop and promote motor function rehabilitation in stroke patients.

Based on the “CPC closed-loop” rehabilitation theory that Jia proposed previously (Jia, 2016), Jia’s team have explored novel application paradigms for stroke rehabilitation. In 2018, we stimulated the spastic muscle groups of the upper limbs after stroke through repetitive peripheral magnetic stimulation (rPMS) and found that rPMS could reduce the spastic state of the upper limb muscles in patients and observed central brain wave changes by electroencephalography (EEG; Chen et al., 2020b). In the same year, we used tDCS combined with upper limb functional electrical stimulation training and found that this intervention technique, which is based on closed-loop rehabilitation theory, could promote the rehabilitation of upper limb motor function in stroke patients (Shaheiwola et al., 2018). Another main application paradigm is camera-based mirror therapy visual feedback (camMVF), which has been proved to enhance limb and brain functions for stroke recovery. In 2018, we also verified the effect of camMVF for improving upper limb function after stroke (Ding et al., 2018). In 2019, we further determined that camMVF-based priming could improve the motor and daily functions of stroke and enhance brain network segregation (Ding et al., 2019). In 2021, we found that camMVF had a priming effect on robot-assisted training to facilitate rehabilitation for people with stroke (Rong et al., 2021). Additionally, we put forward the concept of “associated MT” (Zhuang et al., 2021), a novel paradigm based on camMVF, which could achieve a bimanual cooperation task under camMVF circumstances.

Considering the closed-loop BCI, we have performed several studies to explore its feasibility and clinical and sub-clinical efficacy. Since 2012, we have tested the effects of BCI in the recovery of upper limb motor function and cognitive function after stroke (Li et al., 2012a,b). In 2013, we found that neurofeedback-based BCI could improve the upper limb motor function of stroke patients and enhanced the event-related desynchronization (ERD) intensity of the ipsilesional hemisphere (Li et al., 2013). During 2014 and 2015, we reported some BCI schemes and strategies (Li et al., 2014; Liu et al., 2014; Xia et al., 2015). After that, in 2016, we again explored the clinical effects of electrical stimulation-based and exoskeleton-based BCI on stroke patients (Chen et al., 2016; Li et al., 2016). In 2017, we reviewed the application progress of BCI in hand functional rehabilitation of stroke patients (Jia, 2017). In 2018, we proposed a fast way to detect BCI-inefficient users by using physiological features from EEG signals (Shu et al., 2018b). In 2019, we used tactile stimulation to enhance BCI performance and peripheral magnetic stimulation to decrease upper limb spasticity to expand the scope of BCI application (Shu et al., 2018a; Chen et al., 2020b). Moreover, in 2020, we confirmed the clinical efficacy of BCI training on stroke patients with upper limb dysfunction in both sub-acute and chronic stages, and we explored the closed-loop brain activation changes in sensorimotor rhythm (Chen et al., 2020a; Miao et al., 2020). In 2021, we proposed an inter- and intra-subject transfer calibration scheme for improving feedback performance of the closed-loop BCI training (Cao et al., 2020). In the same year, we compared the differences between motor attempt and motor imagery tasks, which are commonly used in a closed-loop BCI system (Chen et al., 2021). In 2022, we further demonstrated the relationships between sensorimotor rhythm during motor attempt/imagery tasks and upper limb motor impairment in stroke (Chen et al., 2022), which may support the clinical application of the closed-loop BCI system. To sum up, we have explored the closed-loop application of BCI in both the clinic and brain region activations and will move forward to examining its closed-loop brain mechanism.

### Clinical significance of central–peripheral–central closed-loop rehabilitation theory (stroke)

Numerous studies have found that the efficacy of combined intervention techniques based on the closed-loop theory of CPC rehabilitation is significantly greater than that of central- or peripheral-only interventions, which provides new ideas for the rehabilitation of motor dysfunction in stroke.

First, closed-loop neuromodulation can be tailored to each person’s brain function. The CPC closed-loop rehabilitation theory allows for consideration of individual variability in the excitability and connectivity of brain networks. Second, the time course of dynamic changes amid brain function reorganization during stroke rehabilitation based on the closed-loop technology can be taken into account (Grefkes and Ward, 2014). Third, since the modifiability of neurons and networks is a function of their recent activity, which critically determines the direction, extent, and duration of plasticity in neural networks (Müller-Dahlhaus and Ziemann, 2015). This can be used to time the stimulation appropriately by applying a closed-loop brain stimulation method.

### Introduction of possible mechanisms of central–peripheral–central closed-loop rehabilitation

Central interventions can improve synaptic plasticity around the injured brain regions and increase the efficiency of synaptic remodeling, while peripheral interventions may induce synapse formation while promoting the establishment of functional synapses. The closed-loop rehabilitation theoretical technique formed by the organic combination of both can further strengthen synaptic plasticity and the remodeling ability through positive feedback, thus promoting functional recovery.

The CPC closed-loop may function on the basis of the brain’s neuronal plasticity. In the context of closed-loop BCI training, the Hebbian theory is a typical neural mechanism used to explain the changes in the neural system.

Emerging studies about “closed-loop” neuroscience have increased in recent years. “Closed-loop” refers to the complex brain feedback loops and sensorimotor interactions between the brain and environment (Zrenner et al., 2016). As the biofeedback of neural activity, neurofeedback is the basis of “closed-loop” neuroscience, which provides participant neural activation feedback for self-regulation (Sitaram et al., 2017). “Closed-loop” rehabilitation strategies—for example, non-invasive brain stimulation (NIBS), which directly stimulates the brain—have become hot interventions for people with brain injuries, such as stroke (Hummel and Cohen, 2006; Kemps et al., 2022).

Combination strategies are more prevalent in modern rehabilitation than solo interventions. In the present review, based on “closed-loop” neuroscience, we put forward a “CPC closed-loop” rehabilitation strategy, which stresses the use of neurofeedback as a part of the multimodal intervention or adjuvant therapy. The “CPC closed-loop” rehabilitation strategy is a comprehensive intervention to restore neural repair during brain injury rehabilitation and facilitate the best limb function recovery possible. Animal studies have revealed that, after motor cortex injury, forelimb grasping training in rats could increase the projection from the injured cortex to the anterior horn of the spinal cord (Okabe et al., 2016). Exercise intervention can improve the activity of glial cells; strengthen the coupling between astrocytes, microglia, and neurons; and enhance the plasticity of neural function (Li et al., 2021). In addition, central interventions, such as tDCS (Elsner et al., 2020) and repetitive transcranial magnetic stimulation (rTMS; Kirton et al., 2008), have been proven to activate limb function in people with stroke. Hence, the rational combination of “central intervention” with “peripheral intervention” to form a closed-loop intervention model may further enhance limb function and improve the ability of synaptic plasticity. Based on the neurofeedback principle and combination strategies, we assume there are three closed-loop rehabilitation modes for brain injury recovery: large, small, and tiny closed-loop modes.

### Closed-loop rehabilitation system

#### Large closed-loop rehabilitation mode

**FIGURE 2:**
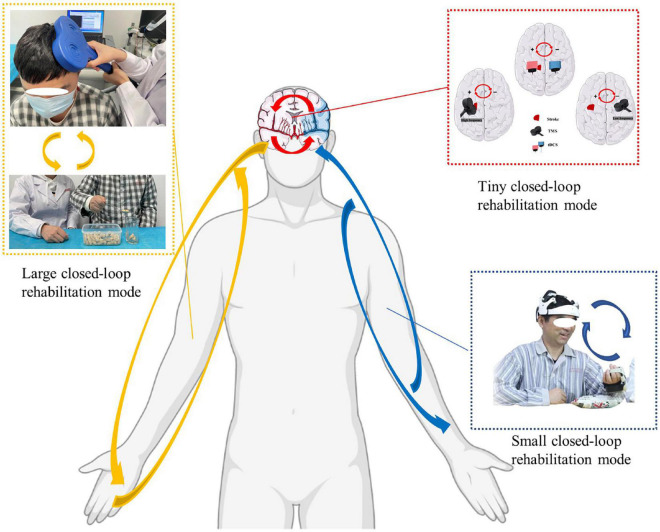
Closed-loop rehabilitation modes: large closed-loop rehabilitation mode, small closed-loop rehabilitation mode, and tiny closed-loop rehabilitation mode (The written informed consent was obtained from the individual for the publication of the image). Red arrow: tiny closed-loop rehabilitation mode, the modulation effect in the intra-hemisphere or the inter-hemisphere. Blue arrow: small closed-loop rehabilitation mode, an intervention strategy that relies on independent, comprehensive intervention that does not combine with peripheral interventions. Yellow arrow: large closed-loop rehabilitation mode, the different combinations of central intervention techniques combined with peripheral intervention technique.

The closed-loop rehabilitation theory can organically combine traditional peripheral interventions and central interventions to form a 2-way transmission, which can select the appropriate treatment mode according to the degree of motor dysfunction of the patient. The complex composition of central and peripheral interventions increases the complexity of closed-loop rehabilitation intervention techniques guided by the closed-loop rehabilitation theory, and the different combinations of central intervention techniques combined with peripheral intervention techniques form new intervention techniques. Based on this, the large closed loop may undergo various forms of central and peripheral combinations, such as tDCS combined with task-oriented training, tDCS combined with functional electrical stimulation of the upper limbs, TMS combined with task-oriented training techniques, TMS combined with peripheral neuromuscular magnetic stimulation techniques (Figure 2), BCI combined with task-oriented training techniques, MT combined with upper limb task-oriented training techniques, MI combined with task-oriented training techniques, and other combinations.

For example, we used a closed-loop rehabilitation technique of transcranial direct current combined with functional electrical stimulation, and the Fugl-Meyer assessment for the upper extremity (FMA-UE) score, muscle tone modified Ashworth scale score, and Broetz hand function test result of upper limb motor function of stroke patients were significantly improved compared to those of the sham stimulation group; specifically, the mean value of FMA-UE was improved by 8.53 points vs. 4.60 points in the sham group, while the mean value of the Broetz hand function test was improved by 11.93 points vs. 6.33 points in the sham group, effectively improving patients’ ability to perform activities of daily living and significantly shortening their inpatient rehabilitation period (Shaheiwola et al., 2018).

In a randomized controlled clinical trial using mirror therapy combined with task-oriented training, the technique protocol significantly improved upper limb motor function and functional independence compared to non-use of the closed-loop rehabilitation intervention technique. Specifically, the upper limb motor ability was improved by 17 points vs. 8.6 points in the conventional rehabilitation group, while functional independence was improved by 17.1 points vs. 6.2 points in the conventional rehabilitation group, with rehabilitation effects of 25.8 and 13.6%, which were 12.7 and 8.7% greater than the effects of conventional rehabilitation (Ding et al., 2019). The above study confirms that techniques based on the closed-loop rehabilitation theory help stroke patients to recover motor function.

#### Small closed-loop rehabilitation mode

The “closed loop” involves actions leading to consequences (future inputs into the brain) that are observable. Unlike the large closed-loop rehabilitation mode, the small closed-loop rehabilitation mode is an intervention strategy that relies on independent, comprehensive intervention that does not combine with peripheral interventions. This intervention can stimulate brain and limb function simultaneously. Moreover, unlike the tiny closed-loop rehabilitation mode, the intervention-based small closed-loop rehabilitation mode generally needs to test and guide patients before treatment to make them familiar with and cooperate with the training process. A critical aspect of the treatment is recommending that patients imagine actively and control their movements and feelings by using multimodal inputs like vision and hearing. The procedure also involves some cortical brain areas and brain networks, such as the prefrontal lobe and attention network (Deconinck et al., 2015). Therefore, small closed-loop strategies such as brain–machine interfaces (BMIs) and mirror visual feedback (MVF) generally require sensory priming and are system-regulation processes.

Many studies have regarded the BMI as a closed-loop device for patients requiring neurology rehabilitation, such as those with a spinal cord injury (Jackson and Zimmermann, 2012). By integrating proprioceptive and visual feedback into the BMI, assistive devices, such as computers and robotic prosthetics, can be controlled by patients. People with paralysis using BMI can learn to control multiple neurons so that external devices and communication can be facilitated, which provides a therapeutic benefit by enhancing voluntary recruitment of surviving motor pathways (Birbaumer and Cohen, 2007; Daly and Wolpaw, 2008; Sitaram et al., 2017). There is promising evidence of BMI efficacy for people with stroke. In previous studies, we found that a BCI (Figure 2) with exoskeleton feedback was practical in sub-acute stroke patients, and patients who presented increasingly stronger or continuously strong activations (ERD) may obtain better motor recovery (Chen et al., 2020a). Further, the motor attempt task may provide better BCI accuracy but has similar activations in the cortex as the motor image task (Chen et al., 2021).

Another small closed-loop strategy is MVF, also called MT. In the past 20 years, MVF has emerged as a powerful tool to facilitate the recovery of disordered movement and to activate underactive brain areas after stroke (Pollock et al., 2014; Thieme et al., 2018; Zhuang et al., 2021). A mirror is placed in the median sagittal plane between 2 limbs, and the mirror side reflects the unaffected limb to avoid direct observation of the affected side. Participants are requested to move their bilateral limbs as far as possible while concentrating on the mirror side. Through this process, mirror visual illusion can be induced to activate the cortical cortex by MVF. Although MVF affects the sensorimotor cortex, the underlying specific mechanism of MVF is still unknown (Deconinck et al., 2015). Saleh et al. (2017) found that MVF could mediate contralesional parietal cortex modulation over the ipsilesional primary motor cortex in chronic stroke patients more effectively compared to the veridical feedback condition, which indicated the existence of network neurofeedback of MVF. MVF is a small closed loop that connects limb activities and brain activation. Based on a closed-loop strategy, we previously designed a novel camera-based MVF, through which participants could receive multiple sensory inputs. One of our studies revealed that camera-based MVF could improve motor recovery, daily function, and brain network segregation in sub-acute stroke patients (Ding et al., 2019).

#### Tiny closed-loop rehabilitation mode

A tiny closed-loop rehabilitation mode usually works on its own and leads to changes in the brain. The changes may exist in 1 of the 2 hemispheres of the brain, thus inducing intra-hemisphere neural plasticity. Changes existing in both hemispheres cause inter-hemisphere neural plasticity. Here, the “closed loop” can be explained as the modulation effect in the intra-hemisphere or the inter-hemisphere. Stimulations from both electricity and magnets can contribute to a closed-loop modulation effect on the brain.

Passive brain stimulation technologies, such as the tDCS and the TMS, are the main ways to form a tiny closed-loop rehabilitation mode (Figure 2). By applying an anode electrode and a cathode electrode on the brain, tDCS is able to activate or inhibit a hemisphere or specified brain region. As for tDCS, an inhibition from the cathode electrode and activation from the anode electrode form an inter-hemisphere modulation by upregulating the excitability of a hemisphere and downregulating excitability of the other hemisphere. By applying high- or low-frequency energy on the brain, TMS is able to activate or suppress the brain hemispheres. An inhibition comes from a low-frequency dosage, while an activation comes from a high-frequency dosage. This can also induce an inter-hemisphere change between the left and right hemispheres.

This phenomenon is usually detected by radiological technology like functional magnetic resonance imaging and electrophysiological techniques like EEG. Recent advances combining TMS with EEG are able to promote new brain stimulation protocols that are controlled by the EEG signal and thus “close the loop” around the brain in a very direct way, short-circuiting the motor–sensory loop (Bergmann et al., 2012).

## Intervention means of closed-loop rehabilitation

### Central interventions

Central nervous system (CNS) intervention is the technique that acts on the brain to modulate neuroplasticity, which plays an important role in promoting functional recovery after stroke. According to the active or passive form of patient participation, it can be divided into intrinsic and extrinsic central interventions. Intrinsic central interventions include MI, MT, and BCI, which require patients to actively issue instructions in the brain to activate the corresponding brain areas and circuits that promote neural remodeling. Extrinsic central intervention is further divided into invasive brain stimulation and NIBS. The former usually requires invasive operations on the patient, such as deep brain stimulation.

Due to the inconvenience of the deep brain stimulation operation, NIBS is more commonly applied in clinical practice. tDCS and TMS are typical methods of NIBS. tDCS can directly affect the excitability of neurons through currents, while TMS can generate reverse induced currents in the cortex by altering the magnetic field, balancing the excitability of the left and right hemispheres, and promoting functional remodeling. However, they cannot directly stimulate deep brain regions. TUS makes up for the defect and provides the possibility of precise intervention in deep brain regions, using the ultrasound energy to stimulate brain tissue, which leads to a series of biological effects that promote recovery after stroke.

Non-invasive brain stimulation has been proven to modulate the process of neuroinflammation in stroke. Researchers (Zhang et al., 2020) found that tDCS (500 μA, 15 min, cathodal) could reduce high levels of neuron-specific enolase, caspase-3, and the Bax/Bcl-2 ratio in middle cerebral artery occlusion rats, which thereby contribute to the resistance of apoptosis and the inhibition of the activation of microglia and astrocyte at the acute phase of ischemic stroke. Furthermore, tDCS treatment significantly decreased the levels of pro-inflammatory cytokines such as interleukin (IL)-1β (Regner et al., 2020), IL-6 (Zhang et al., 2020), and tumor necrosis factor (TNF)-α (Callai et al., 2022) and increased the levels of anti-inflammatory cytokines such as IL-10 (Zhang et al., 2020) in cerebral ischemic penumbra, which can inhibit the neuroinflammatory response in cerebral ischemic penumbra and produce neuroprotective effects in the early stage of stroke. rTMS can significantly mitigate blood–brain barrier (BBB) permeabilization by preserving important BBB components from photothrombotic-induced degradation and decrease peripheral immune cell recruitment and infiltration to the peri-infarct cerebral vasculature by the downregulation of certain cytokines (CXCL10, CD54, CXCL9, and CCL5) (Zong et al., 2020). TUS can also inhibit the activation of microglia and astrocytes by normalizing the expression of inflammatory cytokines such as nuclear factor kappa B, TNF-α, and IL-1β (Zhou et al., 2021). Thus, the CNS interventions can ameliorate the neuroinflammation of stroke, which is induced by both CNS immunity and peripheral immunity.

### Peripheral interventions

Peripheral interventions are a series of rehabilitation treatments that act on the trunk and limbs. They are mainly based on the natural recovery process after CNS injury and follow the general laws of neurodevelopment to promote the functional reconstruction of patients with CNS injury through repetitive training and enhanced motor control. Peripheral intervention techniques include neurodevelopmental techniques, such as Bobath, Brunnstrom, proprioceptive neuromuscular facilitation, and Rood techniques, and also include task-oriented training (TOT), functional electrical stimulation (FES), constraint-induced movement therapy, assistive technology, biofeedback therapy, and rehabilitation robots. These peripheral interventions promote CNS plasticity by continuously feeding sensory information to the CNS through external stimulation and reinforcing the correct motor patterns. However, single peripheral interventions are no longer sufficient to meet the rehabilitation needs of the growing number of patients with CNS injuries, and thus the intrinsic mechanisms and their application in combination with central interventions should be continuously explored. Studies (Zhang et al., 2013) have shown that exercise-based peripheral interventions can reduce the inflammation after reperfusion by inhibiting the activation of microglia and reactivating astrocytes, which subsequently reduce the expression of pro-inflammatory cytokines. It was also found that peripheral electrical stimulation promoted the resolution of ischemic edema and enhanced astrocyte activity in the marginal and distal septal regions of the infarct foci (Park et al., 2021).

### Application of central–peripheral–central closed-loop rehabilitation in cerebral injury

**FIGURE 3:**
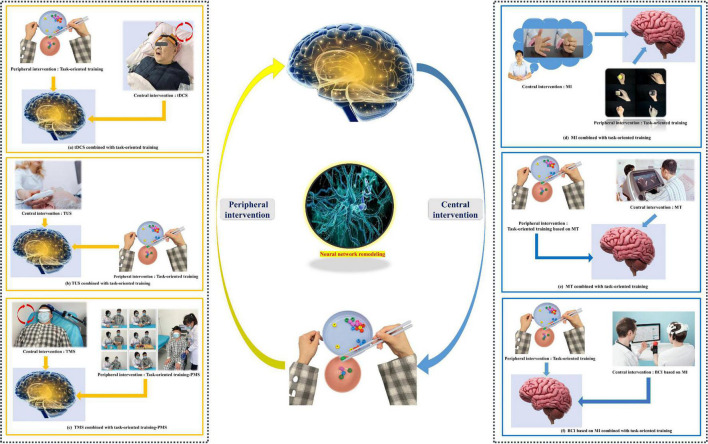
Common paradigms of the “closed-loop” rehabilitation strategy. **(a)** tDCS combined with task-oriented training. **(b)** TUS combined with task-oriented training. **(c)** TMS combined with task-oriented training-PMS. **(d)** MI combined with task-oriented training. **(e)** MT combined with task-oriented training. **(f)** BCI based on MI combined with task-oriented training. (Written informed consent was obtained from the individual for the publication of the image).

The bulk of the research has proved that CPC closed-loop rehabilitation is more effective than single central or peripheral therapy in managing post-stroke dysfunctions, such as motor impairment, aphasia, and dysphagia, and treatment options include tDCS + FES, tDCS + electromyographic biofeedback, tDCS + TOT, rTMS + TOT, and so on (Wang et al., 2012; Baroni et al., 2022; Figure 3). This is reflected in both physiological indicators and clinical manifestations, including motor evoked potentials, the modified Ashworth scale, the Fugl–Meyer motor function assessment, the water drinking test, and so on (Shaheiwola et al., 2018; Muhle et al., 2021). In previous research, we found that tDCS combined with FES is more effective in improving upper limb function in severe chronic stroke patients than sham tDCS combined with FES (Shaheiwola et al., 2018). The effectiveness of this intervention paradigm was further validated by Salazar et al. (2020), who indicated that tDCS plus FES improved the movement cycle time, mean reaching velocity, and handgrip force of chronic post-stroke survivors with moderate or severe impairment. In addition, tDCS combined with FES gait training improved post-stroke patients’ gait regularity better than a FES gait training intervention only (Mitsutake et al., 2021). In the treatment of stroke, some scholars have used our theoretical method (Yu et al., 2020; Yang et al., 2021). There is no best recommendation for CPC therapy. The combination therapy schemes used in animal experiments and clinical trials can be divided into the following categories: central intervention combined with conventional rehabilitation therapy, NMES, TOT, use of a rehabilitation robot, or acupuncture (Li et al., 2018; Xu et al., 2018; Zhao et al., 2022). These findings provide the evidence and potential of the CPC closed-loop theory.

In addition to synergistic therapy, central–peripheral combined therapy can also play a role in precise targeting and adjustment of stimulation time. In recent years, using EEG-based BCI technology to navigate other intervention technologies has gradually become a research hotspot. The current technology combinations are as follows: (1) EEG + rTMS, where rTMS stimulation target positioning is guided by task-state EEG analysis; (2) EEG/magnetoencephalography + tDCS, where EEG or magnetoencephalography tracking is used to guide timing and stimulus settings for tDCS; and (3) EEG + TUS, where navigation is performed through EEG to guide stimulation targets for TUS. In addition, therapeutic paradigms that guide central intervention by analyzing the periphery are also being explored.

## Neuroimmunological mechanisms

**FIGURE 4:**
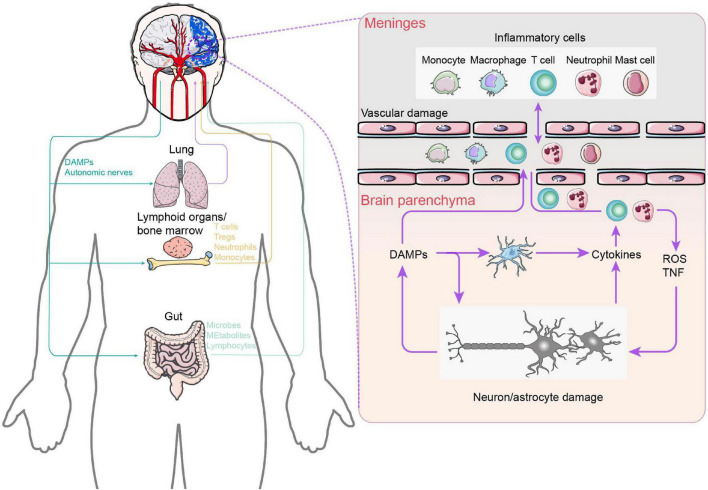
Central and peripheral immune response crosstalk in stroke. After ischemia, necrotic neuronal cells appear and release DAMPs due to intracellular adenosine triphosphate depletion and hypotonic hypoxia. On the one hand, DAMPs activate innate immune receptors on brain resident immune cells, leading to the release of cytokines and chemokines, which, in turn, promote additional neutrophil entry. Neutrophils damage the brain by producing reactive oxygen species and TNF. On the other hand, brain-derived DAMPs leak into the circulation and activate systemic immunity, mobilizing innate immune cells in lymphoid organs, the lungs, and the gut. Circulating peripheral immune cells subsequently extravasate into the brain parenchyma and meninges. In addition, the increase in gut permeability triggers bacteria and their metabolites to enter into the brain parenchyma. In the left part: Purple: Lung–Brain axis. Yellow: Lymphoid organs / bone marrow–Brain axis. Purple: Gut–Brain axis.

Neuroplastic alterations or functional reorganization mediated by interhemispheric competition and vicariation models are the well-known recovery mechanisms of post-stroke rehabilitation. Numerous studies have established conclusively that the cerebral cortex displays spontaneous phenomena of neuroplasticity during brain injury (Dimyan and Cohen, 2011; Alia et al., 2017; Khan et al., 2017; Dabrowski et al., 2019). The disruption of neural networks does stimulate a reorganization of synaptic junctions that is highly sensitive to the appearance of damage (Li and Carmichael, 2006). Nevertheless, this reorganization suffers from the oversimplified or even incorrect rationale for CPC closed-loop rehabilitation due to limited beneficial effects after stroke. Actually, activation of brain-resident cells, such as microglia and astrocytes, and blood-borne immune cells, including periphery monocytes/macrophages and T lymphocytes, as well as the immunoreactive molecules they secrete are quickly engaged at the onset of brain injury (Iadecola et al., 2020). The crosstalk between the peripheral and CNS immune components mentioned above significantly correlates with functional recovery in patients with ischemic brain injury and stroke (Figure 4). More importantly, the immune response plays a bidirectional role in functional recovery in both the acute and chronic phases after stroke. Therefore, it is critical to understand the mechanisms of immune activation following stroke in order to implement rehabilitation interventions accordingly to different stages of disease in the CPC closed-loop rehabilitation. Here, we examine the role of CNS immunity and its complex interaction with peripheral immunity in closed-loop rehabilitation.

### Central and peripheral immune response crosstalk

Immediately following cerebral ischemia, microglial activation occurs before necrotic neuronal cell appears due to intracellular adenosine triphosphate depletion and hypotonic hypoxia (Rupalla et al., 1998). Microglia features increased arborization, exploratory behavior, and ameboid transformation to prepare for phagocytizing dead cells and neutrophils. Subsequently, necrotic cells in the cerebral ischemia core secrete damage-associated molecular patterns (DAMPs) into the extracellular circulation to further activate brain resident immune cells mainly composed of microglia and astrocytes (Benakis et al., 2014). On the one hand, microglia suppress post-stroke inflammation by producing anti-inflammatory cytokines (IL-10, transforming growth factor β) and the neurotrophic factor IGF-1, removing cellular debris by phagocytosis and suppressing astrocyte activation, thus promoting angiogenesis and tissue re-organization (Lalancette-Hebert et al., 2007; Kawabori et al., 2015; Otxoa-de-Amezaga et al., 2019). On the other hand, cytokine profiles [IL-12 and interferon-γ for type 1 T helper (Th1) cells; IL-6, transforming growth factor β, and IL-23 for type 17 T helper (Th17) cells] secreted by activated microglia induce the generation of Th1 and Th17 cells to promote neuroinflammation (Wan, 2010; Martinez-Sanchez et al., 2018). Meanwhile, interferon-γ mainly produced by Th1 cells and IL-17 mainly produced by Th17 cells induce microglia to express IL-1β, IL-6, and TNF-α, which in turn induce the generation of Th1 and Th17 cells (Watanabe et al., 2016). In addition, stroke-induced activation of the sympathetic and parasympathetic nervous systems may mediate immunodepression after stroke. Ischemic injury immediately activates the sympathetic nervous system, leading to the contraction and shrinkage of peripheral immune organs (Dorrance and Fink, 2015). The parasympathetic nervous system antagonizes the pathways that are activated by the sympathetic nervous system and then is suppressed following stroke. Some studies have demonstrated that splenic contractions prompt peripheral immune cells to migrate into the brain injury (Ajmo et al., 2008; Fathali et al., 2013). Thus, splenectomy prior to ischemic stroke or irradiation of the spleen following stroke significantly reduces infarct size as well as the number of neutrophils and activated microglia in the brain.

### Gut–brain axis

For decades, researchers have studied the relationship between the gastrointestinal (GI) tract and the brain. The “gut–brain axis” refers to the specific linkage between the GI tract and the CNS, which consists of a bidirectional exchange between them. In other words, through the gut–brain axis, the gut and the brain communicate with each other (Socała et al., 2021). From an organ perspective, the brain represents the center and the gut represents the periphery. The brain–gut axis may profile the closed-loop pathway of the CPC theory.

Inflammatory signaling occurs in both the afferent (“gut-to-brain”) and efferent (“brain-to-gut”) directions across the gut–brain axis to relay the host’s health status and stimulate regulatory responses that help to restore homeostasis or amplify inflammation in a context-dependent manner. Since the GI tract is in direct contact with antigens, intestinal microorganisms and their metabolites derived from food and the environment, in addition to the existence of physical barriers such as the gut–vascular barrier (Spadoni et al., 2015), the intestinal tract is also the place where the human body has the largest number of immune cells (Mowat and Agace, 2014). In the intestine, the innate and adaptive immune systems work together to respond quickly to intestinal damage *via* specific immune cell types, such as M cells (Lai et al., 2020), macrophages (Muller et al., 2014), mast cells (Reed et al., 2003), ILC2 cells (Klose et al., 2017), ILC3 cells (Talbot et al., 2020), B-cells (Rojas et al., 2019), CD4 T-cells (Yan et al., 2021), and CD8 T-cells (White et al., 2018). Besides immune cells, neurons and glial cells in the enteric nervous system also participate in intestinal immunity, and their dysfunction will alter the normal intestinal–brain communication and the control of the CNS over the intestine (Huh and Veiga-Fernandes, 2020). Under normal physiological conditions, the CNS is distinguished from its peripheral environment by the BBB. In addition, the CNS also contains a certain number of immune cells, such as microglia (Erny et al., 2015), astrocytes (Rothhammer et al., 2016), and natural killer cells (Sanmarco et al., 2021). Although meninx monocytes, neutrophils, and some subsets of B-cells are supplied directly from the skull and spinal marrow, these CNS-related immune cells are mainly derived from the periphery of the CNS (Cugurra et al., 2021). Complex interaction networks are formed between these immune and non-immune cells to regulate the inflammatory responses in the CNS and the GI tract (Agirman et al., 2021).

Recent studies have shown that the gut–brain axis regulates the allowed homeostasis of the body by mediating the transmission of inflammatory signals, which play an important role in an array of inflammatory diseases (Agirman et al., 2021). The transmission of inflammatory signals in the intestine–brain axis is bidirectional, and they can transmit inflammatory signals through 3 parallel but interconnected pathways: the systemic–humoral pathway, the cellular immune pathway, and the neuronal pathway. There is growing evidence that the gut microbiome is a major environmental factor that shapes the brain through the microbiome–gut–brain axis (Kelly et al., 2017; Mayer et al., 2022). This new perspective on gut and brain interactions has also been applied to the pathophysiology of several brain disorders that were previously attributed solely to pathophysiological processes that occurred within the brain. Calorie restriction provided long-term stroke rehabilitation benefits, in part by modulating gut microbiota (*Bifidobacterium* enrichment), which suggests the possibility of obtaining a favorable outcome in long-term stroke rehabilitation by fecal microbiota transplantation from calorie restriction–treated donors or *Bifidobacterium* supplementation (Huang et al., 2021). Research has shown that specific changes in the cecal microbiota of the Peptococcaceae and the Prevotellaceae are associated with the degree of injury in mice with brain injury. These effects are mediated by norepinephrine released by the autonomic nervous system and alter the production of cecal mucin and the number of goblet cells (Houlden et al., 2016). In addition, post-stroke gut microbiota dysbiosis promotes the proliferation of Th1 and Th17 cells in the intestine as well as the migration of gut-derived T-cells and monocytes to the ischemic brain, exacerbating neuroinflammation (Singh et al., 2016). As a bidirectional modulating system, it forms a closed-loop neuroimmune mechanism between the brain and the gut *via* neuroanatomical, immunological, and neuroendocrine pathways.

### Lung–brain axis

The physiological changes caused by the interaction of microbial endocrinology and the external environment affect not only affect the gut but also the lungs (Bajinka et al., 2020). According to the findings of a few new studies, the lung microbiome may have an impact on the CNS. Five potential mechanisms are known or predicted, as follows: direct microorganism translocation, effects of lung microbes on systemic immunity, nerves, the hypothalamic–pituitary–adrenal axis, and metabolic changes (Bell et al., 2019). This results in the formation of a closed-loop potential mechanism involving the lungs and brain.

The CNS autoimmune process is not only dependent on nerve tissue but also influenced by peripheral organs. According to research, smoking and pulmonary infection significantly increase the risk of developing multiple sclerosis (Olsson et al., 2017). Furthermore, previous research has shown that T-cells capable of causing CNS autoimmune reactions migrate into lung tissue before entering the CNS, where they settle and develop into pathogenic effector cells and long-term memory cells (Odoardi et al., 2012). Pulmonary microbiota disorders have a significant impact on CNS autoimmune responses. According to new research, using neomycin to transform the lung microbiota into a lipopolysaccharide-producing bacterial taxa can transform microglia into the gene-expression state of the type I interferon pathway, significantly inhibiting the pro-inflammatory response and relieving autoimmune symptoms (Hosang et al., 2022).

In the current study, focal ischemic stroke altered the respiratory pattern, caused histological lung damage and inflammation, and reduced the phagocytic capability of alveolar macrophages while leaving the pulmonary function unchanged. The mechanism underlying reduced phagocytic capability of alveolar macrophages appears to be related to serum release rather than BALF mediators. Furthermore, IL-6 gene expression was increased in macrophages and endothelial cells but not in epithelial cells isolated from stroke animals’ lungs. These findings suggest the occurrence of dynamic crosstalk between the brain and lungs even after relatively mild/moderate brain injury caused by a stroke (Samary et al., 2018). A closed-loop pattern of the CPC might explain the potential link between the lungs and brain, but the mechanism of the central intervention to regulate lung function through neuroimmunology and peripheral interventions to regulate brain injury through changes in lung function is not clear for patients with brain injury.

## Current challenges and future prospects

Closed-loop rehabilitation takes full advantage of central and peripheral intervention techniques that are applied simultaneously or sequentially to patients with brain injury, achieving a “1 + 1 > 2” synergistic therapeutic effect. Central interventions, such as MT and BCI—especially emerging non-invasive brain stimulation techniques (TMS, tDCS, and TUS)—facilitate the development of closed-loop rehabilitation. Numerous studies have further revealed the common mechanisms at play, including synaptic plasticity and functional reorganization mediated by the interhemispheric competition and vicariation models. Furthermore, we place emphasis on the role of CNS immunity and its complicated crosstalk with the peripheral immunity in closed-loop rehabilitation. This crosstalk has particular salience in post-stroke dysfunction, which triggers both beneficial and harmful immune processes. A major frontier in stroke research concentrates on understanding these interactions in order to develop new strategies to prevent and reduce the burden of stroke. Future work will focus on delineating precise clinical strategies for closed-loop rehabilitation based on non-invasive brain stimulation.

In addition, it would be reasonable to modulate the immune system toward beneficial post-stroke rehabilitation by precise non-invasive stimulation in view of data suggesting that this improves clinical outcomes. Furthermore, post-stroke immunodepression puts the patient at higher risk of infection, and clinical treatment strategies should be adjusted accordingly. Finally, the closed-loop rehabilitation of patients with stroke may be ameliorated by advances in specific areas, including exploration of whether modulating immune circuits can reduce the incidence of massive nerve damage or nerve cell death during acute stroke, whether immunity plays a role in different closed-loop systems, and whether bidirectional interventions to prevent post-stroke immunodepression or hyperimmune activation can reduce the risk of infection so as to avoid autoimmune responses against the brain. It is conceivable that future advances in bidirectional interventions will provide in-depth knowledge of closed-loop rehabilitation and that individualized brain stimulation will allow for notable enhancements in rehabilitation success.

We are remarkably sanguine that multimodal and personalized closed-loop rehabilitation will be part of the future of stroke and other brain diseases. Large, small, and tiny closed-loop rehabilitation modes can satisfy the treatment of different stages of disease accordingly, but more studies are needed to confirm which closed-loop mode best matches which stage of the disease. Note that further study on the mechanism will be more conducive to the clinical promotion of the system, especially in the area of the immune system. Additionally, future advances in non-invasive closed-loop systems should make rehabilitation interventions feasible and accessible to large numbers of individuals. Ideally, the non-invasive closed-loop technologies will have the ability to modulate precise brain region at millimeter spatial resolutions and in deep brain nuclear applications. Rather than TMS and tDCS, TUS accompanied by high spatial resolution and deep transcranial penetration can be tailored to the patient’s specific pathophysiology and disease severity, and then tracked by neuroimaging tools in real time. It is with such technological breakthroughs and an in-depth understanding of modulating mechanisms that we hope that this novel closed-loop rehabilitation will flourish to successfully improve the quality of life of patients with brain diseases.

## Author contributions

The author confirms being the sole contributor of this work and has approved it for publication.
